# Retrograde axonal transport of poliovirus and EV71 in motor neurons

**DOI:** 10.1016/j.bbrc.2022.08.015

**Published:** 2022-08-10

**Authors:** Seii Ohka, Soon Hao Tan, Shohei Kaneda, Teruo Fujii, Giampietro Schiavo

**Affiliations:** a Neurovirology Project, Tokyo Metropolitan Institute of Medical Science, 2-1-6, Kamikitazawa, Setagaya-ku, 156-8506, Tokyo, Japan; b Department of Pathology, University of Malaya, Lembah Pantai, 59100, Kuala Lumpur, Malaysia; c Institute of Industrial Science, The University of Tokyo, Meguro-ku, 153-8505, Tokyo, Japan; d Department of Neuromuscular Diseases, Queen Square Institute of Neurology, University College London, London, WC1N 3BG, United Kingdom; e UCL Queen Square Motor Neuron Disease Centre, University College London, London, WC1N 3BG, United Kingdom; f UK Dementia Research Institute, London, WC1N 3AR, United Kingdom

**Keywords:** EV71, Poliovirus, Axonal transport, Transgenic mice, Microfluidic devices

## Abstract

Poliovirus (PV) can spread through neural pathway to the central nervous system and replicates in motor neurons, which leads to poliomyelitis. Enterovirus 71 (EV71), which is closely related to PV, is one of the causative agents of hand-foot-and-mouth disease and can cause severe neurological diseases similar to poliomyelitis. Since PV is similar to EV71 in its motor neurotoxicity, we tried to understand if the results obtained with PV are of general applicability to EV71 and other viruses with similar characteristics. Using microfluidic devices, we demonstrated that both PV capsid and the PV genome undergo axonal retrograde transport with human PV receptor (hPVR), and the transported virus replicated in the soma of hPVR-expressing motor neurons. Similar to PV in hPVR-transgenic (Tg) mice, neural pathway ensuring spreading of EV71 has been shown in adult human scavenger receptor class B, member 2 (hSCARB2)-Tg mice. We have validated this finding in microfluidic devices by showing that EV71 is retrogradely transported together with hSCARB2 to the cell body where it replicates in an hSCARB2-dependent manner.

## 1 Introduction

Both poliovirus (PV) and enterovirus 71 (EV71) are non-enveloped RNA viruses classified into human *Enterovirus* [1,2]and are neurotropic, causing severe neurological diseases in humans. PV is a causative agent of poliomyelitis, resulting in flaccid paralysis [3,4]. EV71 is a causative agent of hand-foot-and-mouth disease in infants and young children [5], but it occasionally leads to brain-stem encephalitis and acute flaccid paralysis [6].

After injection into calf muscles, PV enters the sciatic nerve and causes an initial paralysis of the inoculated limb in transgenic (Tg) mice expressing the human PV receptor (hPVR/CD155) [7,8]. PV disseminates through the sciatic nerves of hPVR-Tg mice by fast retrograde axonal transport and causes paralysis in a hPVR-dependent manner. During axonal transport, PV is transported in organelles containing hPVR [9,10]. The PV genomic RNA is assumed to be transported with the capsid as an intact particle through the axon, undergoing uncoating only upon arrival in the cell body. However, direct evidence showing that PV genome is transported with capsid through axons is currently lacking.

The neural pathway of mouse-adapted EV71 has been previously suggested to occur in non-Tg suckling mice [11]. However, sciatic nerve transection did not reduce virus spreading from hindlimbs to the CNS, casting doubts on the presence of this route in non-Tg mice. Moreover, mouse-adapted EV71 has different characteristics from ordinary EV71. On the other hand, Tg mice expressing the functional EV71 receptor, human scavenger receptor class B, member 2 (hSCARB2) [12] are susceptible to ordinary EV71 [13], though the neural pathway has not been elucidated to date.

Since PV shares motor neurotoxicity with EV71, we aimed to address whether the results obtained with PV are of general applicability to EV71 and other viruses with similar characteristics. Here, we report that both PV capsid and genome undergo axonal retrograde transport in motor neurons (MNs) together with hPVR, and that retrogradely-transported PV replicated in the soma of hPVR-MNs. Similarly, EV71 was retrogradely transported and replicated in MNs in an hSCARB2-dependent manner both in mice and primary MNs, where hSCARB2 undergoes axonal retrograde transport in the presence of anti-hSCARB2 antibodies.

## 2 Materials and methods

### 2.1 Cells

HeLa S3 cells were cultured in suspension [10]. Mouse L929 and human rhabdomyosarcoma (RD)-hSCARB2 cells (a kind gift of Dr. K. Fujii) were cultured similarly to Vero cells [13]. RD-hSCARB2 cells were supplemented with 4 µg/ml puromycin (Calbiochem).

### 2.2 Viruses

Purified PV type 1 Mahoney strain PV1(M)OM [14] or EV71 strain SK-EV006/Malaysia/97 (SK-EV006; genogroup B) [15] were used as a representative of PV or EV71, respectively. Virus purification was performed as previously described [10,12], and virus titer was determined by median tissue culture infectious dose (TCID_50_) in Vero cells. Experiments using pathogens were approved by the Committee for Experiments using Recombinant DNA and Pathogens at the Tokyo Metropolitan Institute of Medical Science (TMiMS).

### 2.3 Fluorescent double-labeling of PV

PV RNA genome labeling with SYTO82 was done according to Ref. [16] during the virus amplification process [10]. After 140 min incubation of HeLa S3 cell suspension with the virus at 37 ° C, SYTO82 was added to the cell suspension at a final concentration of 25 µM and kept in the dark thereafter. After additional 4 h incubation at 37 °C, cells were collected and the virus purified. Capsid labeling with AlexaFluor488 was performed according to Ref. [10]. Double-labeling did not significantly affect viral titer.

### 2.4 Antibodies

The following primary antibodies were used: anti-PV rabbit antibodies (absorbed by fixed mouse L929 cells), anti-hSCARB2 goat antibodies (AF1966, R&D), anti-EV71 polyclonal antibodies (kindly provided by H. Shimizu, NIID, Japan) [17], and control goat IgG antibodies (AB-108-C, R&D). AlexaFluor488, 568, and 647 donkey, or goat anti-rabbit or goat IgG (H + L) (Life Technologies) were used as secondary antibodies.

### 2.5 Mice

The ICR or C57BL/6CrSlc (Japan SLC, Inc.) were used as non-Tg mice for PV or EV71, respectively, together with the ICR-PVRTg21 [18] or hSCARB2-Tg10 [13] mouse strains. Four week-old mice were injected intramuscularly with EV71. Embryonic day 13 fetuses were used for MN primary cultures [19]. Experiments using mice were approved by the Animal Use and Care Committee of TMiMS and performed in accordance with the Guidelines for the Care and Use of Animals.

### 2.6 Transection of sciatic nerve and intramuscular injection with EV71

Transection of the sciatic nerve and viral intramuscular injection was carried out as previously described [8]. Briefly, under anesthesia, a 5 mm long nerve section of the sciatic nerve was removed. Up to 5 µl of viral suspension was intramuscularly inoculated into the left calf 2 cm from the transection point in 1–4 sites.

### 2.7 Primary culture of MNs in microfluidic devices

Cultures of MNs in microfluidic devices with 450 µm-long microgrooves were carried out as previously described [19].

### 2.8 Virus infection

Five days post-plating, after axons had extended across the microgrooves, cells were treated with virus in the somatic or axonal compartment [19]. The wells of the axonal compartment were inoculated at a multiplicity of infection (MOI) of 3 TCID_50_ of viruses except for MOI of 4000 TCID_50_ of PV for real-time imaging. The levels of the medium in the virus-containing compartment were kept lower than in the opposite compartment, thus completely preventing diffusion of the virus through the microgrooves. At 0, 6, 10, 14, 19 and 24 h after infection (h.a.i.) for hPVR-Tg MNs axonal compartment infection with PV, at 0, 6, 10 and 24 h.a.i. for hPVR-Tg MNs somatic compartment infection with PV, at 0, 14, 19 and 24 h.a.i. for non-Tg MNs infection with PV, at 0, 14, 19 and 24 h.a.i. for hSCARB2-Tg infection with EV71 and at 0, 16, 24, 48 h.a.i. for non-Tg infection with EV71, 10 µl medium per well was collected, and then the virus titer determined.

### 2.9 Axonal transport imaging

Fluorescent PV was added into the axonal compartment of hPVR-Tg MNs cultured in a microfluidic device, which was placed on the stage of an inverted microscope. Cells were incubated at 37 °C with 5% CO_2_ in an environmental chamber during live imaging. Axonal transport was imaged by DeltaVision Personal DV (Cytiva). Images were analyzed by Imaris (Carl Zeiss Microscopy) and Image J software (National Institutes of Health, USA) [20].

### 2.10 Immunofluorescence

Immunofluorescence was carried out as previously described except for the fixation and mounting medium [9]. MNs were fixed in PBS (−) containing 4% paraformaldehyde and 20% sucrose for 10 min at room temperature. After staining, cells were mounted in Vectashield with DAPI Mounting Medium (Vector Laboratories). Samples were imaged with a laser-scanning microscope (TCS SP2, Leica Microsystems). Images were analyzed by Imaris and Image J [21].

### 2.11 Statistical analysis

Statistical analysis was performed using GraphPad Prism 7.00 (GraphPad Software Inc.).

## 3 Results

### 3.1 Both PV RNA genome and capsid undergo axonal retrograde transport in MNs with hPVR

**Fig. 1:**
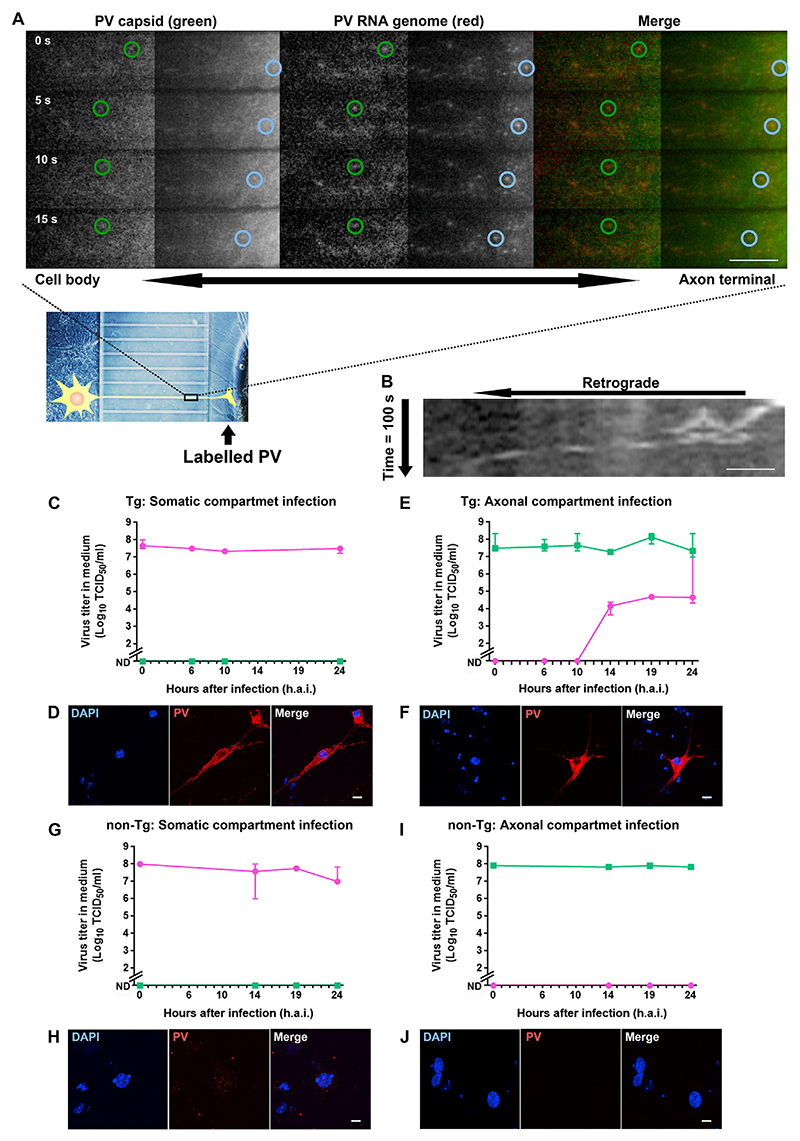
PV RNA genome is transported retrogradely with capsid in MNs expressing hPVR (A) Double-labeled PV was added to the axonal compartment followed by observation under fluorescent microscope. AlexaFluor488-labeled capsid and SYTO82-labeled RNA genome are in green and red, respectively. Green and blue circles indicate the relative position of two moving axonal organelles. The time in second after starting the observation is shown. The brightness of the left of the images was enhanced. Scale bar, 10 µm. (B) Kymographs for the organelle marked by the blue circles. Scale bar, 1 µm. (C to J) After the addition of PV to either the somatic or axonal compartment of MNs cultured in a microfluidic device, the medium was collected at the indicated time points followed by titration. Pink and green indicate titer in somatic and axonal compartments, respectively. Error bars indicate SD. (C, D) Somatic compartment infection of hPVR-Tg MNs. (E, F) Axonal compartment infection of hPVR-Tg MNs. (G, H) Somatic compartment infection of non-Tg MNs. (I, J) Axonal compartment infection of non-Tg MNs. (D, F, H, and J) Representative images of somatic compartment at 6 h.a.i. Scale bars, 5 µm. (C, D, G to J) n = 3. (E, F) n = 2 for 14 and 19 h.a.i., n = 4 for 6 and 10 h.a.i., and n = 6 for 0 and 24 h.a.i. (For interpretation of the references to colour in this figure legend, the reader is referred to the Web version of this article.)

Organelles containing both capsid-labeled PV and hPVR are retrogradely transported from axon terminals to cell bodies in MNs [10]. Although it has been hypothesized that the PV RNA genome is transported with the capsid as a complete infectious particle and start uncoating in the cell body, this has not been conclusively demonstrated. To test this hypothesis, PV RNA genome and capsid were labeled with SYTO82 and AlexaFluor488, respectively. Double-labeled PV was added to the axonal compartment of MNs expressing hPVR cultured in microfluidic devices [19]. Virus transport was then monitored by time-lapse fluorescence microscopy (Fig. 1A and B). From around 7 min after infection, green PV capsids could be easily identified in axons and were found to undergo axonal retrograde transport together with red PV genomes. Rate of transport ranged between 0.075 µm/s (blue) and 0.15 µm/s (green), with maximum velocities of 1.28 µm/s (green). These transport speeds are compatible with those observed for cytoplasmic dynein [22]. These results suggest that intact PV particles are retrogradely transported prior to uncoating.

### 3.2 Retrogradely transported PVs replicate in the soma of hPVR-expressing MNs

We then addressed the fate of PV viral particles entering MNs at axon terminals or in the cell body by exploiting the fluidic separation ensured by microfluidic devices. MNs grown in microfluidic devices were treated with PV in the axonal or somatic compartments, and the viral titer in the medium determined (Fig. 1C–J). When MNs expressing hPVR were infected with PV in the somatic compartment, the viral titer in this compartment was in the order of 10^7^ TCID_50_ until 24 h.a.i. In contrast, the virus was not detected in the axonal compartment (Fig. 1C). However, when MNs expressing hPVR were treated with PV in the axonal compartment, a viral titer over 10^4^ TCID_50_ was detected in the somatic compartment starting from 14 h.a.i., which was stable until 24 h.a.i. (Fig. 1E). The viral titer in the axonal compartment was maintained around 10^7^ TCID_50_ until 24 h.a.i. These results suggest that PV is retrogradely transported and replicates in the cell body after infection in the axonal compartment, whereas the virus is not transported anterogradely to axon terminals and/or does not replicate in axon terminals after somatic infection. PV antigen was detected in the cytoplasm at 6 h.a.i. by immunofluorescence in both somatic and axonal compartment infection (Fig. 1D, F). These results show that infection with PV in the soma and axon terminals lead to virus replication in the cell body.

PV is retrogradely transported upon binding to hPVR [10]. To confirm the essential role of hPVR in the axonal retrograde transport of PV in MNs, MNs lacking hPVR were used in parallel and treated with PV either in the somatic or axonal compartment (Fig. 1G–J). Interestingly, the viral titer in the somatic compartment remained very low at least until 24 h.a.i. after adding the virus to the axonal compartment (Fig. 1I), indicating that the virus does not undergo retrograde transport to the cell body and/or does not replicate in the absence of hPVR. These results confirm that hPVR is essential for the axonal retrograde transport of PV and its replication in MNs. PV antigen was not detected in the cytoplasm at 6 h.a.i. upon somatic and axonal infection (Fig. 1H, J). These results show that treatment of MNs lacking hPVRs with PV in both somatic and axonal compartments do not lead to virus replication.

### 3.3 A neural transmission pathway through the sciatic nerve is present in hSCARB2-Tg mice

**Fig. 2:**
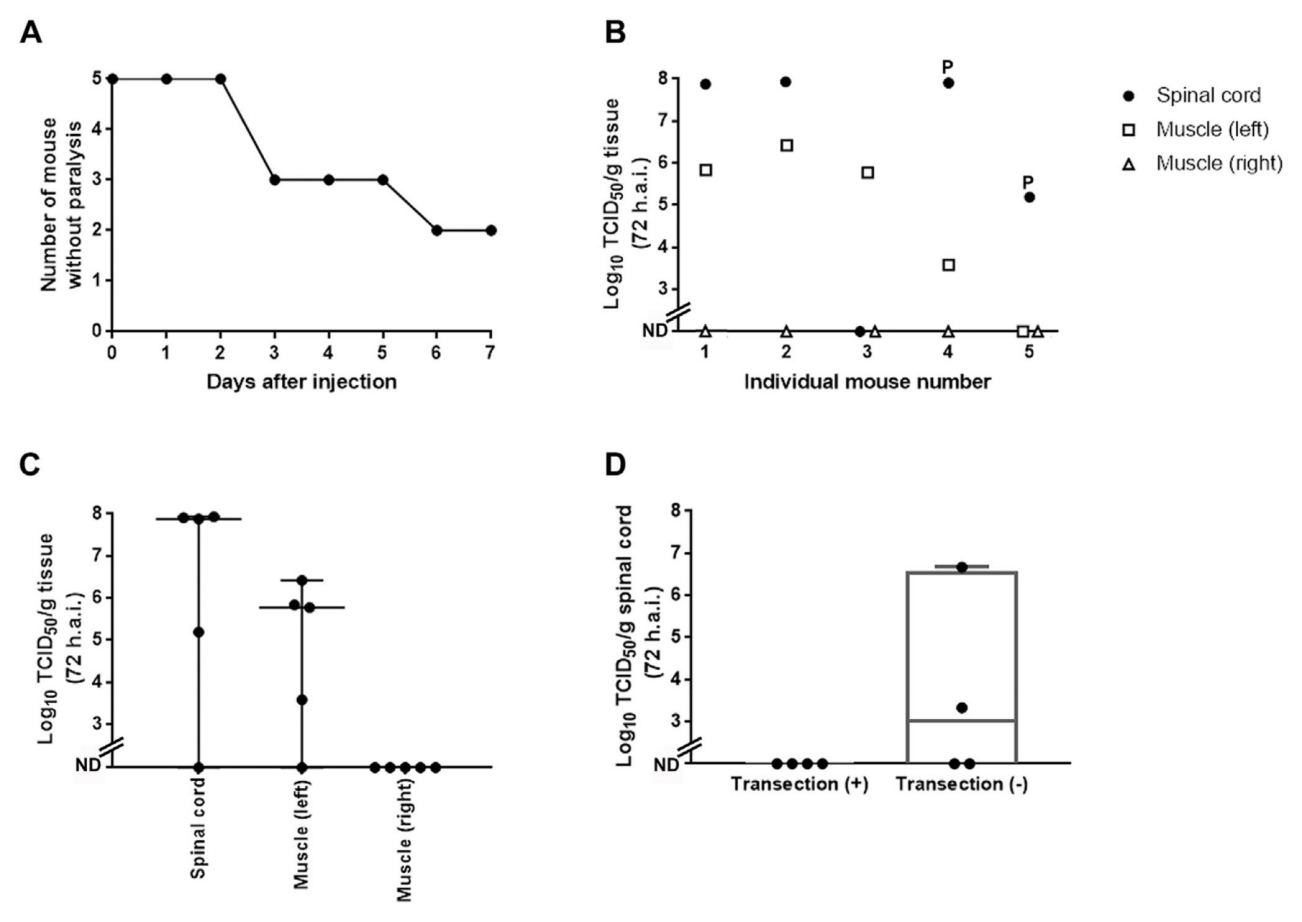
EV71 is retrogradely transported through the sciatic nerve in hSCARB2-Tg mice (A) Number of non-paralysis time course after intramuscular injection with EV71 (n = 3). Repeated experiments showed the same trend. (B) Virus titer in spinal cord or muscle (left or right) 72 h after intramuscular injection with EV71. Filled circle, square, and triangle indicate spinal cord, muscle (left), and muscle (right), respectively. Number shown under horizontal axis indicates the mouse identification number. Mouse 4 and 5 showed paralysis at 72 h.a.i., indicated as P in the graph. (C) Viral titer in spinal cord or muscle (left or right) at 72 h.a.i from experiment shown in B. Each circle indicates one mouse. (D) Viral titer in spinal cord 72 h after intramuscular injection with EV71. Prior to the viral injection, sciatic nerve transection or sham operation were carried out. Each circle indicates one mouse. Median and range are shown in C and mean and range are shown in D.

To examine whether a neural transmission pathway plays a role in non-mouse-adapted EV71 infection in adult hSCARB2-Tg mice, 5.5 × 10^6^ TCID_50_ EV71 was intramuscularly injected into four-week old hSCARB2-Tg mice, which were monitored for appearance of flaccid paralysis in the injected limb (Fig. 2A). Three days after injection, two out of five mice showed overt signs of paralysis, whereas an additional mouse showed paralysis on day six. The appearance of limb paralysis implies that the virus was transported from axon terminals to MN cell bodies in the spinal cord, leading to replication of the virus and halting of MN activity. To further investigate this route of viral transmission, the EV71 titer in the spinal cord or muscle were examined at 72 h.a.i. (Fig. 2B and C). In the spinal cord, four out of five mice showed significant high titer of the virus (Fig. 2B and C). Two of them showed paralysis on the injected limb on 72 h.a.i., whereas other mice did not, despite the high titer of the virus in the spinal cord (Fig. 2B). These results suggest that in mice injected intramuscularly, the virus replicates in the spinal cord, leading to paralysis of the injected limb. In the muscles of the injected side (left), four out of five mice showed high virus titers, whereas no virus was detected on the contralateral (right) side (Fig. 2B and C). These results suggest that blood-borne virus does not start replication outside the injected area for at least 72 h.a.i. and that the high EV71 titer in the spinal cord is due to its direct transfer through the sciatic nerve.

To prove that a sciatic nerve-mediated neural pathway is implicated in EV71 infection and symptomatology, the sciatic nerve was transected or sham-operated before ipsilateral intramuscular injection of EV71, followed by virus titer determination in the spinal cord at 72 h.a.i. (Fig. 2D). In the sham operated mice, virus titer in spinal cord was detected in two out of four mice, whereas none of the mice with transected sciatic nerve displayed EV71 in the spinal cord (Fig. 2D). These results suggest that sciatic nerve transection halted virus transfer into the spinal cord and its replication.

### 3.4 Retrogradely transported EV71 replicates in the soma of hSCARB2-expressing MNs

**Fig. 3:**
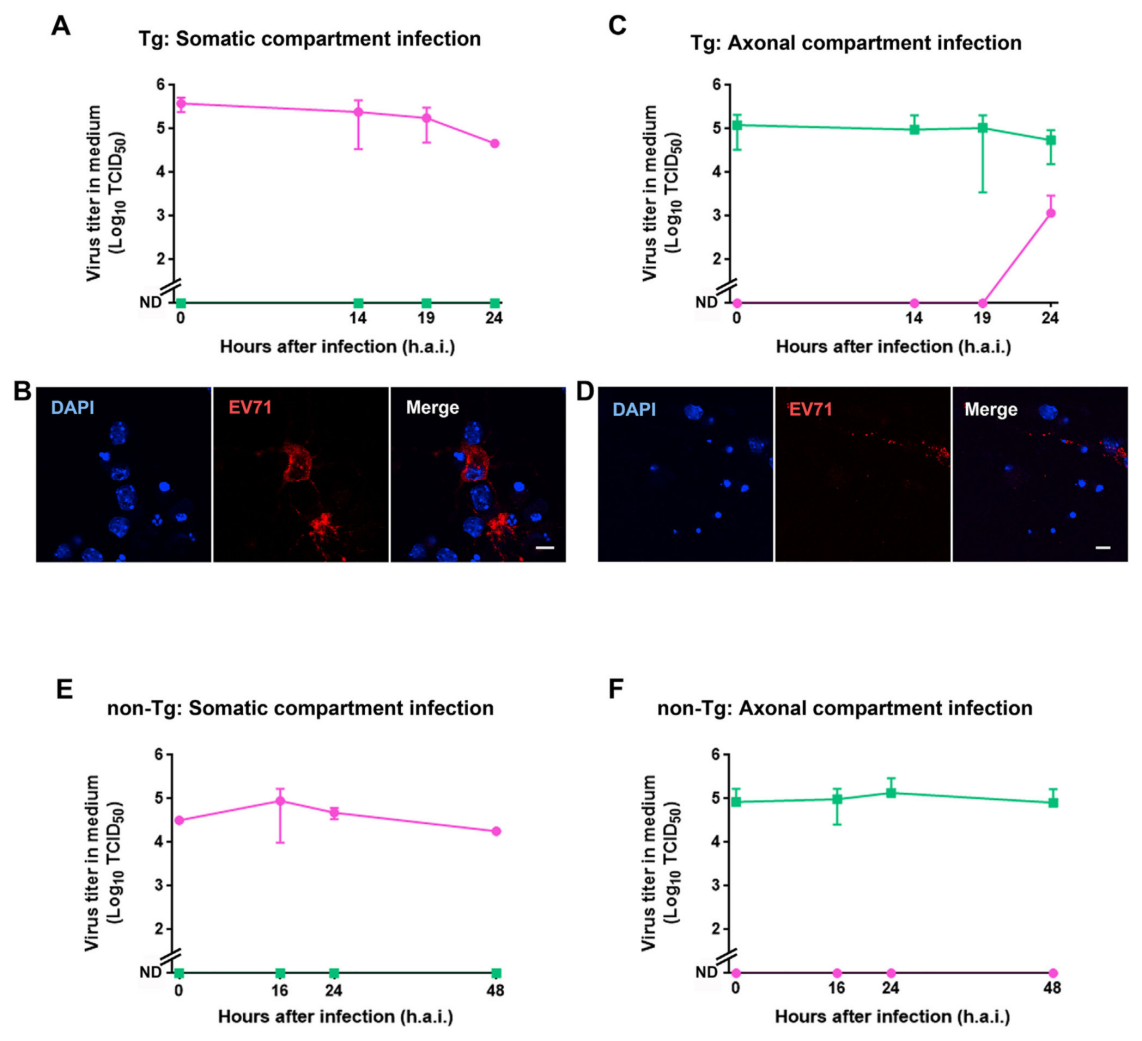
EV71 is retrogradely transported in MNs expressing hSCARB2 After addition of EV71 in either the somatic or axonal compartment of MNs cultured in a microfluidic device, the medium was collected at indicated time points followed by titration. (A) Somatic compartment infection of hSCARB2-Tg MNs. (C) Axonal compartment infection of hSCARB2-Tg MNs. (B, D) Representative immunofluorescence images of somatic compartment at 8 h.a.i. Infection of non-Tg MNs in the somatic (E) and axonal (F) compartment. Scale bars, 5 µm.

To conclusively demonstrate the retrograde transport of EV71 in MNs, we utilized microfluidic devices similar to those used for PV in Fig. 1C–J. When MNs expressing hSCARB2 were incubated with EV71 in the somatic compartment, the virus titer remained relatively constant until 24 h.a.i., whereas no virus was found in the axonal compartment (Fig. 3A). In contrast, when MNs expressing hSCARB2 were infected with EV71 in the axonal compartment, the virus titer in the somatic compartment reached 10^3^ TCID_50_ at 24 h.a.i. (Fig. 3C). EV71 antigen was detected in the cytoplasm at 8 h.a.i. in both cases (Fig. 3B, D). These results suggest that virus is retrogradely transported to the cell body after entering the axon, and replicates in the soma, whereas the virus is not transported and/or replicate in axon terminals after addition to the somatic compartment.

To investigate the contribution of hSCARB2 towards the axonal transport of EV71, primary MNs not expressing hSCARB2 were infected as described above (Fig. 3E and F). Adding EV71 to the axonal compartment did not result in an appreciable appearance of the virus in the somatic chamber (Fig. 3F), indicating that the virus is not transported and/or replicate in the soma in the absence of hSCARB2. These results confirm that hSCARB2 is essential for the axonal retrograde transport of EV71 and its replication in primary MNs.

### 3.5 hSCARB2 is retrogradely transported in hSCARB2-Tg MNs

**Fig. 4:**
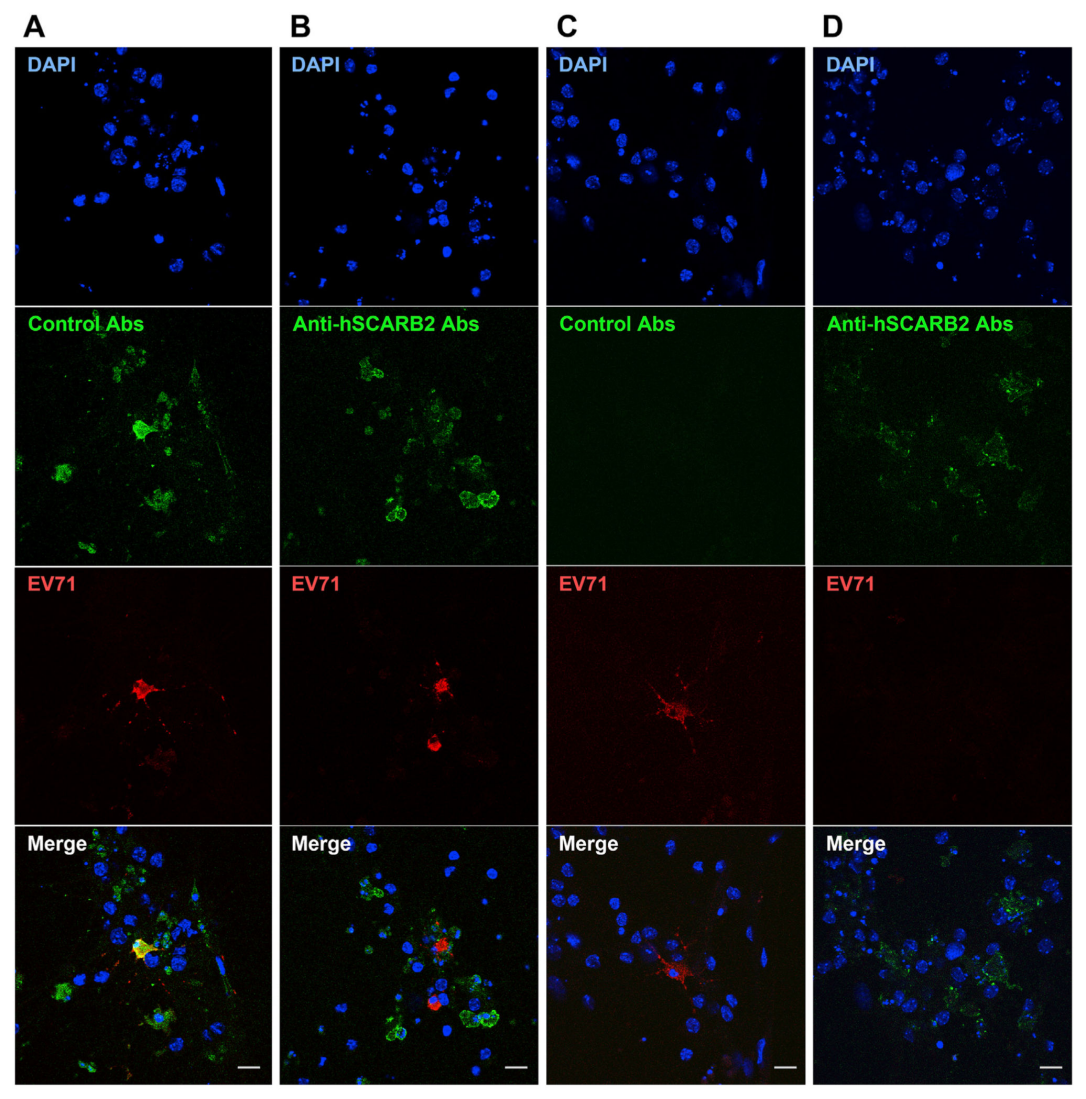
hSCARB2 is retrogradely transported in MNs expressing hSCARB2 hSCARB2-Tg MNs were infected with EV71 in the presence of control IgG (A, C) or anti-hSCARB2 antibodies (B, D) added to the somatic (A, B) or axonal (C, D) compartment. Cells were fixed 16 h.a.i. followed by immunofluorescence. Representative images show DAPI in blue, control and anti-hSCARB2 antibodies in green, whereas EV71 is in red. Scale bars, 5 µm. (For interpretation of the references to colour in this figure legend, the reader is referred to the Web version of this article.)

hSCARB2 is necessary for retrograde axonal transport and/or replication of EV71. To further confirm hSCARB2 contribution for EV71 axonal transport and/or replication, 0.5 mg/ml of anti-hSCARB2 or control antibodies were added to either compartment of MNs cultured in a microfluidic device followed by EV71 infection (5.6 × 10^3^ TCID_50_). Transport or anti-hSCARB2 antibodies and EV71 was assessed by immunofluorescence (Fig. 4). When anti-hSCARB2 antibodies and EV71 were added to the somatic compartment, their specific signal was detected in cell bodies after 16 h.a.i. (Fig. 4A and B). The number of EV71-positive cells with control antibodies was 123 at 16 h.a.i., whilst it decreases to 50 in the presence of anti-hSCARB2 antibodies. These results imply that the anti-hSCARB2 antibodies inhibit viral infection. Strikingly, when control antibodies and EV71 were added to the axonal chamber, no antibodies were detected in cell bodies (Fig. 4C). However, when anti-hSCARB2 antibodies and EV71 were added to the same compartment, antibody immunoreactivity was detected in cell bodies without EV71 signal at 16 h.a.i. (Fig. 4D). These results suggest that anti-hSCARB2 antibodies undergo efficient axonal retrograde transport to the MN soma upon binding to hSCARB2. When antibodies and EV71 were added to the axonal chamber, the number of EV71-positive cells with control antibodies was 3 at 16 h.a.i. (4 at 24 h.a.i.), whilst no EV71-positive cells were observed upon treatment with anti-hSCARB2 antibodies in the same experimental conditions. Although the number of EV71 positive cells is small, these results suggest that anti-hSCARB2 antibodies are protective and efficiently inhibit EV71 axonal transport and/or its replication.

## 4 Discussion

Although a neural pathway responsible for the spread of mouse-adapted EV71 was hypothesized in non-Tg suckling mice [11], no evidence is currently available to support a similar route of spreading for ordinary EV71 in adult susceptible mice. Here, we report such evidence in mature hSCARB2-Tg mice. In these experiments, high titers of the virus are required to ensure the smallest possible volume for intramuscular injection. However, the propensity of EV71 to aggregate [23] make it difficult to achieve such high titers, which may lead to poor reproducibility. In our tests, not all the mice showed muscle paralysis. Nevertheless, about 50% of mice showed paralysis, with 80% of them showing high virus titer in the spinal cord at 72 h.a.i. The median of the virus titer in the spinal cord was higher than that found in the injection area, indicating that the high titers of EV71 in the spinal cord at 72 h.a.i. is likely to be due to efficient replication. No virus was detected in right muscle at 72 h.a.i., indicating that the virus derived from viremia would not start replicating until after 72 h.a.i. Transection of the sciatic nerve completely inhibited virus replication in the spinal cord at least for 72 h.a.i. in all the mice intramuscularly injected with the virus, indicating that the virus detected in the spinal cord at 72 h.a.i. is delivered via the sciatic nerve. These results strongly suggest that a neural pathway for non-mouseadapted EV71 transmission exists in mature hSCARB2-Tg mice.

Interestingly, Scarb2 was found in the proteome of signaling endosomes, a class of organelles undergoing axonal retrograde transport [24], which is enriched in markers of neurological diseases. This finding, together with our result demonstrating the axonal retrograde transport of hSCARB2, suggest that signaling endosomes play a fundamental role in the spreading of EV71 from muscles to the CNS and consequent paralysis.
